# Characteristics of dermal fibroblasts and their biotherapeutic value in musculoskeletal system diseases

**DOI:** 10.3389/fbioe.2026.1821137

**Published:** 2026-06-08

**Authors:** Li Zhou, Qin Zhang, Hao Yang, Guicheng Zhang, Ming Fan, Rongguang Jin, Shen’ao Zhou

**Affiliations:** 1 FibroX Therapeutics (Shanghai) Inc., Shanghai, China; 2 State Key Laboratory of Cell Biology, Center for Excellence in Molecular Cell Science, CAS, Shanghai Institute of Biochemistry and Cell Biology, Chinese Academy of Sciences, University of Chinese Academy of Sciences, Shanghai, China

**Keywords:** cell therapy, dermal fibroblasts, diseases of the musculoskeletal system, extracellular matrix, immunomodulation, regenerative medicine

## Abstract

As the principal functional cells in the dermal layer of the skin, dermal fibroblasts can not only synthesize and remodel extracellular matrix components such as collagen and elastin, but also secrete a variety of growth factors and exert immunomodulatory functions. They play a pivotal role in maintaining skin structural homeostasis and wound repair, and their unique biological properties also make them a key subject of research in regenerative medicine. Musculoskeletal diseases (such as tendinopathy, fracture and intervertebral disc degeneration) are often accompanied by insufficient local tissue repair capacity, chronic inflammation and extracellular matrix degradation. In recent years, autologous or allogeneic fibroblast transplantation therapy has attracted increasing attention in orthopedic regenerative medicine. A number of clinical trials and basic studies have confirmed the beneficial effects of fibroblasts in promoting cartilage repair, alleviating inflammatory responses, improving tendon healing, and delaying intervertebral disc degeneration. This review systematically examines the sources, phenotypes, and functional characteristics of dermal fibroblasts. It focuses on their therapeutic potential in musculoskeletal system diseases (MSDs), including tendon injury, disc degeneration, and fracture healing. At the same time, it analyzes the challenges faced by current applications and the future directions for development, providing a theoretical reference for their clinical transformation.

## 1 Introduction

Fibroblasts are among the most widely distributed stromal cells in the human body, participating in various physiological processes such as structural support of tissues, synthesis of extracellular matrix (ECM), and wound repair, and extensively supporting the basic functions of organs. Fibroblasts are an extremely heterogeneous cell type, with fibroblasts from different tissue sources exhibiting significant phenotypic and functional specificity, and even differences within the same tissue (Griffin et al., 2020; Lynch and Watt, 2018).

As the largest organ of the human body and the first line of defense against external environments, the skin is home to dermal fibroblasts, which play a crucial role in its physiological and pathological functions (DesJardins-Park et al., 2018; Gurtn et al., 2008). When the skin is damaged (such as by sunburn, cuts, burns or lacerations), dermal fibroblasts play a crucial role in signaling to other key wound cell types and in directly closing and/or repairing the damaged area. It is a complex process (Gurtn et al., 2008). Dermal fibroblasts have gradually become a hotspot in cell therapy research due to their ease of acquisition (via minimally invasive skin biopsy), ease of expansion *in vitro*, minimal ethical controversy, and strong repair capabilities.

Musculoskeletal diseases, such as tendinopathy, osteoarthritis, ligament injury and intervertebral disc degeneration, are one of the leading causes of disability worldwide. They are mainly manifested as chronic pain, functional impairment and reduced quality of life, and severe cases may result in lifelong disability. Taking intervertebral disc degeneration as an example, the global annual incidence of lumbar intervertebral disc degeneration is 5.5%, affecting approximately 403 million people (Ravindra et al., 2018). Meanwhile, the incidence rates of tendinopathy, fractures and osteoarthritis remain high among athletic populations and in aging societies (Aabedi and Agrawal, 2026). Traditional therapeutic approaches, including analgesics, physical therapy and surgical intervention, mostly focus on symptomatic control rather than structural tissue repair, and are unable to reverse or halt disease progression. Therefore, exploring biological therapeutic strategies to promote tissue regeneration and restore functional integrity has become a core research topic in regenerative medicine.

In the maintenance of tissue homeostasis and the repair of musculoskeletal injuries, fibroblasts have long been regarded as “structural supporting cells” that synthesize and remodel the extracellular matrix to provide mechanical support for tissues. However, studies over the past decade have indicated that this understanding is overly simplistic. In fact, fibroblasts represent a highly heterogeneous and functionally diverse cell population. They not only maintain the structural integrity of tendons, ligaments, joint capsules and interstitial spaces of skeletal muscle by secreting extracellular matrix components such as collagen and elastin, but also participate deeply in inflammatory regulation, tissue remodeling and intercellular communication. Under the modulation of an appropriate mechanical microenvironment, fibroblasts exhibit the potential to facilitate tissue repair, suppress excessive inflammation, and even differentiate into a regenerative phenotype (Jac et al., 2024; Amernik et al., 2018; Bouffi et al., 2011).

In recent years, cell therapy has emerged as a new frontier in regenerative medicine. Among various cell types, fibroblasts exhibit great therapeutic potential due to their robust paracrine function and immunomodulatory properties. They have shown encouraging outcomes in animal models of tendon injury, intervertebral disc degeneration, and fracture healing, as well as in early clinical trials. This review systematically elucidates the characteristics of dermal fibroblasts and their therapeutic potential in musculoskeletal diseases.

## 2 Characteristics of dermal fibroblasts

### 2.1 Source and acquisition advantage

Generally, the number of fibroblasts in dense connective tissue is greater than that in loose connective tissue of the same volume. Therefore, the main source of fibroblasts for isolation and culture is dense connective tissues such as dermis (Alt et al., 2011).

The source of material for this part is extensive and relatively easy to obtain, such as discarded foreskin tissue after circumcision or minimally invasive skin tissue from behind the ear, buttocks, or limbs. Compared with bone marrow mesenchymal stem cells (BMSCs) and adipose-derived stem cells (ADSCs), dermal fibroblasts require less donor trauma and can be autografted, thereby avoiding immune rejection. Furthermore, they exhibit strong *in vitro* passaging capacity, enabling rapid acquisition of large numbers of cells to meet treatment needs.

### 2.2 Phenotypic characteristics

#### 2.2.1 Morphology and markers

Fibroblasts were first reported in the 19th century, with German pathologist Rudolf Virchow being the first to describe them as a distinct cell type- “spindle-shaped cells of the connective tissue”. The term “fibroblast” was first coined by Ernst Ziegler to describe the cells that produce new connective tissue after wound healing (Molenaar, 2003; Muhl et al., 2020). Fibroblasts have a typical cellular morphology, appearing as long, spindle-shaped, or stellate cells with abundant cytoplasm and large, flattened nuclei. Fibroblasts are the most common cells in connective tissues throughout the body, and dermal fibroblasts are defined by their origin in the dermal layer of the skin.

Although dermal fibroblasts have been extensively studied, their phenotypic characteristics have not been well characterized, nor are there any specific markers to prove their identity. Previous studies considered FSP1 a protein marker that specifically recognizes fibroblasts and named it Fibroblast-Specific Protein (Strutz et al., 1995). Subsequent research revealed that FSP1 is also expressed in various cell types across different lineages, indicating that it is not “absolutely specific” to fibroblasts (Fendt et al., 2023). However, it remains an extremely important and classic marker of fibroblasts, especially activated fibroblasts. Plikus et al. published an article titled “Fibroblasts: origin, definition, and functions in health and disease” in *Cell*, proposing markers for Pan-fibroblasts derived from human skin, including COL1A1^+^, DCN^+^, CD90^+^, PDGFRα^+^, PDGFRβ^+^, LUM^+^, DCN^+^, MFAP5^+^, Vimentin^+^, Lineage^−^ (CD31^−^CD45^−^E-cad^−^) (Plikus et al., 2021). Dermal fibroblasts exhibit low expression of major histocompatibility complex class II (MHC-II) and costimulatory molecules, and thus their immunogenicity is relatively low. This makes allogeneic transplantation possible and lays the foundation for the development of off-the-shelf cell therapy products (Zhou et al., 2025).

The dermis has distinct layers that are easily identifiable histologically but cannot be distinguished from the skin tissue by mechanical means (Janson et al., 2012). The papillary dermis is a thin layer located beneath the epidermis. In contrast, the underlying reticular dermis is much thicker than the papillary dermis and contains a large amount of fibrous extracellular matrix (Harper and Grove, 1979). Therefore, dermal fibroblasts are divided into two subpopulations based on their anatomical location: papillary dermal fibroblasts and reticular dermal fibroblasts. Papillary dermal fibroblasts are located in the superficial layer of the dermis, are spindle-shaped, have stronger proliferative capacity, and express COL6A5^+^, APCDD1^+^, HSPB3^+^, CD39^+,^ and CD26/DPP4^+/−^. Reticular dermal fibroblasts are located in the deep layer of the dermis, are stellate in shape, proliferate slowly, and express CD36^+^, CTHRC1^+,^ and CD26/DPP4^+/−^ (Philippeos et al., 2018; Sole-Boldo et al., 2020; Vorstandlechner et al., 2020).

**FIGURE 1:**
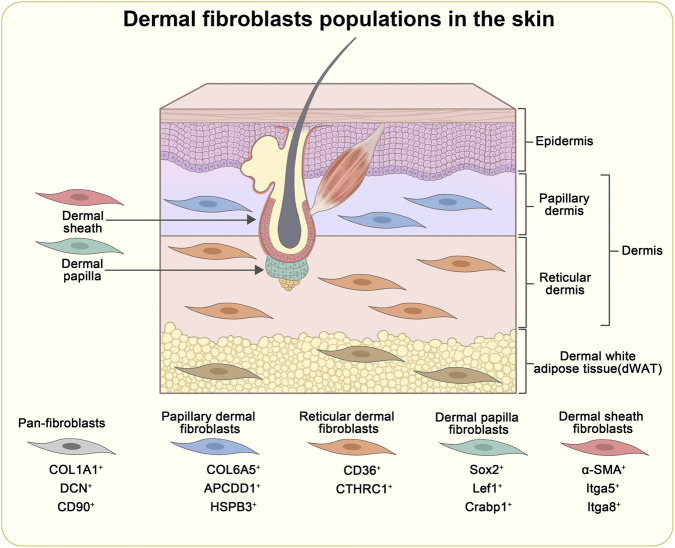
Classification and markers of dermal fibroblasts.

Several stromal cell subpopulations have also been identified at locations closely associated with hair follicles. These follicle-associated fibroblasts develop from papillary dermal condensates through the activation of the WNT and SHH pathways, including dermal papilla fibroblasts (inducing the formation and cycling of hair follicles) expressing Sox2^+^, Lef1^+^, and Crabp1^+^, and dermal sheath fibroblasts (containing self-renewing hair follicle dermal stem cells) expressing α-SMA^+^, Itga5^+,^ and Itga8^+^ (Lim et al., 2018; Talbott et al., 2022). As shown in Figure 1, we have summarized the markers of dermal fibroblasts based on their classification in the skin.

#### 2.2.2 Heterogeneity

Fibroblasts exhibit extensive heterogeneity, and their functional characteristics depend on their organ of origin and location within the body. Fibroblasts derived from different developmental sources and anatomical sites exhibit distinct identification markers. Even though they all originate from the mesoderm, fibroblasts from different anatomical sites and physiological or pathological states exhibit significant heterogeneity. Differences in fibroblast phenotype and function can even be observed in different parts of the same organ (Kendall et al., 2014). For example, fibroblasts in different regions of the dermis (such as the subepidermal papillary dermis and the reticular dermis) exhibit distinct functions and phenotypes. Fibroblasts in the reticular dermis have a weaker proliferative capacity and are more inclined to synthesize collagen. In comparison, fibroblasts in the papillary dermis have a stronger proliferative capacity and are more active in wound repair. Currently, research on the developmental heterogeneity of dermal fibroblasts is relatively thorough. It is generally believed that the differences result from the combined effects of intrinsic factors, such as transcriptional regulatory networks and epigenetic processes, and extrinsic influences, such as cell-cell contacts, secreted signaling factors, and interactions with ECM components. These factors can vary depending on the location of the cells within the body (Lynch and Watt, 2018).

### 2.3 Functionalities

#### 2.3.1 Synthesis and remodeling of the extracellular matrix

**FIGURE 2:**
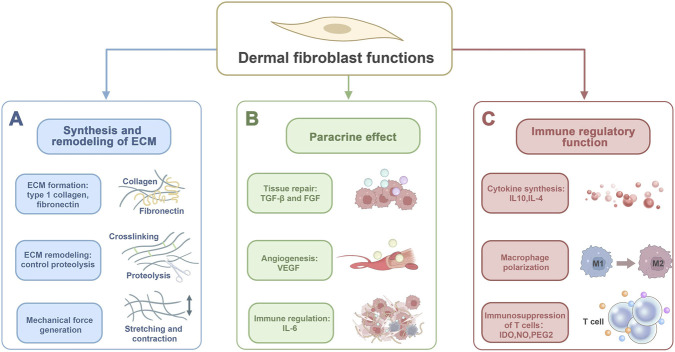
Functions of dermal fibroblasts. **(A)** Dermal fibroblasts produce and maintain the ECM. **(B)** Dermal fibroblasts secrete a variety of cytokines and growth factors, exerting paracrine effects. **(C)** Dermal fibroblasts have immune regulatory functions.

Dermal fibroblasts are important cell types *in vivo* that perform a variety of functions and participate in biological activities (Figure 2). A major common function of fibroblasts is to produce and maintain the ECM (Figure 2A). They produce and secrete structural proteins and adhesion proteins of the ECM, as well as all components of the matrix, providing support, elasticity, and structural integrity to tissues. Structural proteins possess unique properties that can enhance the rigidity (e.g., type 1 collagen) or plasticity (e.g., elastin) of the ECM. Adhesion proteins, like fibronectin and laminin, connect cells to the ECM. At the same time, the matrix supports nutrient entry into tissues inaccessible to blood vessels and serves as a pathway for intercellular communication (Kendall et al., 2014). Silverman et al. using high-resolution live-cell imaging technology, for the first time revealed at the single-cell level that fibroblasts assemble secreted collagen and fibronectin molecules into oriented functional fibers. The study found that when the protrusions of two or more cells contract synergistically, the extracellular matrix molecules between them are subjected to tensile strain. This mechanical process can directly induce conformational changes in fibronectin molecules and drive their assembly into fibers. Subsequently, secreted collagen binds to these fibronectin fibers to form durable composite fibers. In tissues under high mechanical load such as tendons, this mechanically driven matrix assembly mechanism may underlie the formation of functional tissue structures (Silverman et al., 2023). Fibroblasts also actively remodel the ECM microstructure by regulating proteolysis through covalent cross-linking, protein glycosylation, and the actions of modifying enzymes such as lysyl oxidase, matrix metalloproteinases (MMPs), and MMP inhibitors, as well as by balanced secretion (Lu et al., 2011).

#### 2.3.2 Paracrine effect

In addition to producing ECM, dermal fibroblasts also secrete various cytokines and growth factors, including transforming growth factor-β (TGF-β), vascular endothelial growth factor (VEGF), fibroblast growth factor (FGF), and interleukin-6 (IL-6). These factors regulate cell proliferation, differentiation, angiogenesis, and immune response, providing a favorable microenvironment for tissue regeneration (Tomasek et al., 2002; Eckes et al., 2000) (Figure 2B). Hannon et al. elucidated the paracrine regulatory mechanism of the FGF family during skeletal muscle development. FGF-2 can be released from expressing cells and act on neighboring cells in a paracrine fashion to modulate skeletal muscle development (Hannon et al., 1996). Havis et al. systematically investigated the roles of FGF and TGF-β signaling pathways in tendon differentiation using a chick embryo limb development model. FGF synergizes with TGF-β to induce SCX expression, respond to mechanical stimuli, and sustain tendon differentiation (Havis et al., 2016). During tissue injury, they play a role in inflammation and immune cell recruitment, with fibroblasts responding and synthesizing cytokines and chemokines to help guide the immune response (Ogawa et al., 2006).

#### 2.3.3 Immune regulatory function

Dermal fibroblasts have immune regulatory functions, inhibiting excessive inflammatory responses and promoting the transition from an inflammatory environment to a repairing environment (Figure 2C). For example, they inhibit the proliferation of T lymphocytes, regulate macrophage polarization, reduce the secretion of TNF-α and IFN-γ, and increase the secretion of IL-10 and IL-4. This immunosuppression of T cells relies on IFN-γ activation and is mediated by indole 2,3-dioxygenase in dermal fibroblasts (Haniffa et al., 2007; Shimabukuro et al., 1992; Jones et al., 2007). Bouff found that the immunosuppressive effect exerted by dermal fibroblasts can also be mediated by the secretion of NO and PGE2 (Bouffi et al., 2011). Lei’s study revealed the role of the Flightless protein (Flii) secreted by fibroblasts in immune regulation. The results showed that Flii is not only localized intracellularly in fibroblasts but also constitutively secreted extracellularly via the late endosome/lysosome pathway. Secreted Flii can directly bind to LPS, thereby competitively inhibiting TLR4 activation, negatively regulating the inflammatory response of macrophages, and preventing excessive immune injury (Lei et al., 2012).

## 3 The biotherapeutic value of dermal fibroblasts in musculoskeletal system diseases

**FIGURE 3:**
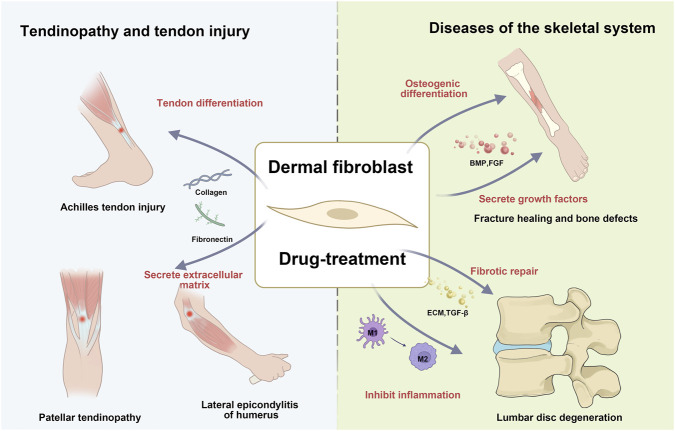
The biotherapeutic value of dermal fibroblasts in musculoskeletal system diseases.

Dermal fibroblasts, owing to their abundant paracrine functions, play important roles in tissue repair and immune regulation and have emerged as promising therapeutic tools in regenerative medicine for musculoskeletal diseases (Figure 3).

### 3.1 Tendinopathy and tendon injury

#### 3.1.1 Achilles tendon injury

The Achilles tendon is a dense connective tissue composed of collagen fibers and tendon cells, exhibiting a spiral structure overall. It lacks blood vessels and has an uneven distribution, making it prone to injury and associated with a poor prognosis. Common causes of Achilles tendon injury include trauma, strain, degenerative changes of the Achilles tendon, uneven stress distribution, and drug use. Due to the constant stress on the Achilles tendon during sports and poor blood circulation, recovery after injury is relatively slow, and tissue adhesion and scar proliferation may occur. Conservative treatment and surgical repair cannot effectively restore the biomechanical properties of the Achilles tendon (Ogunsola et al., 2026). Wei Liu et al. implanted autologous dermal fibroblasts and tendon cells onto polyglycolic acid (PGA) non-woven fabric fibers, respectively, to form a cell scaffold structure. After 7 days of *in vitro* culture, the scaffold was implanted into hybrid pigs to repair a defect of the flexor digital superficial tendon. The results showed that new tendon-like tissue was formed at the implantation site. Histological examination indicated the formation of collagen fibers, including type I and type III. The cells and collagen fibers were arranged along the direction of force, resembling normal tendons. The engineered tendons composed of fibroblasts and tendon cells exhibited tensile strength similar to that of natural tendons, approximately 75% of the strength of natural tendons. It is demonstrated that the application of tissue engineering technology, using dermal fibroblasts as seed cells, can form tendon-like neotissue in animals and repair tendon defects (Liu et al., 2006). Obaid conducted a randomized, double-blind clinical study on the treatment of refractory Achilles tendinopathy with autologous dermal fibroblasts. The study enrolled 40 injured Achilles tendons, and significant differences in VISA and VAS scores were observed between the treatment group and the control group at the 6-month follow-up. The findings preliminarily demonstrated the safety and short-term efficacy of dermal fibroblasts in the treatment of Achilles tendinopathy (Obaid et al., 2012). Notably, this paper has been retracted, and no detailed explanation for the retraction has been provided in public information. This indicates serious concerns regarding the reliability of the study. Its retraction suggests that high-quality clinical evidence to support this approach is currently lacking in the field. Accordingly, research on dermal fibroblast therapy for Achilles tendinopathy remains in the preclinical stage, and further fundamental and clinical studies are required to validate the application value of fibroblasts in the management of Achilles tendinopathy.

#### 3.1.2 Lateral epicondylitis of the humerus

Lateral epicondylitis of the humerus, also known as “tennis elbow”, is a chronic strain disease characterized by localized pain in the lateral epicondylar region of the humerus, aseptic inflammation of the soft tissues surrounding the lateral epicondylar of the humerus caused by acute and chronic injuries, and affecting wrist extension and forearm rotation functions. The etiological factors of humeral lateral epicondylitis include overuse, malposition, improper training, poor circulation, muscle imbalance, etc., (Bongers et al., 2002). This is considered a degenerative process, in which the tendon fails to repair and is replaced by immature repair tissue, resulting in weakened biomechanical properties (Nirschl and Pettrone, 1979). Treatment options for humeral lateral epicondylitis are limited. Conservative treatment can only provide temporary relief of symptoms, and surgical intervention is necessary for intractable epicondylitis. Connell et al. cultivated collagen-producing cells derived from dermal fibroblasts and evaluated the safety and potential use of this cell preparation in the treatment of refractory lateral epicondylitis in a preliminary study. Twelve patients were enrolled in the study, and collagen-producing cells derived from dermal fibroblasts were injected under ultrasound guidance into the total extensor origin site of material tearing and fibrous discontinuity. Six months after treatment, the Patient-Rated Tennis Elbow Evaluation (PRTEE) score, a scale used to assess pain severity and functional impairment, decreased from 78 to 12. Ultrasound imaging revealed a significant reduction in both the number and thickness of tendon tears in the patients. Patients had favorable prognoses (Connell et al., 2009). These preliminary results suggest the safety of injecting collagen-producing cells derived from dermal fibroblasts into patients, and may indicate therapeutic potential for patients with intractable lateral epicondylitis. Although this study provides proof-of-concept evidence for fibroblast therapy in the treatment of lateral humeral epicondylitis, it still has certain limitations, including a small sample size, the absence of a control group, and an overly short follow-up period of only 6 months. Subsequent studies should expand the sample size, prolong the follow-up duration, and adopt a randomized controlled design to generate higher-quality clinical evidence supporting the use of fibroblasts in the treatment of lateral humeral epicondylitis.

#### 3.1.3 Patellar tendinopathy

Patellar tendinopathy refers to inflammation or lesions of the tendon (patellar tendon) connecting the kneecap (patella) to the shinbone (tibia) caused by overuse or injury. It commonly occurs in athletes who frequently perform jumping activities, which is why it is also called ‘jumper’s knee,’ and is primarily characterized by pain at the front of the knee that worsens after exercise. Among adolescent athletes, the incidence of patellar tendinopathy (5.8%) is three times that of Achilles tendinopathy (1.8%). In people aged 50–79 years with no history of knee injury, the incidence of patellar tendinopathy exceeds 28% (Soler et al., 2023). Conventional treatments include conservative therapy (analgesic medication and rehabilitation training) and surgical intervention. Andrew et al. demonstrated that autologous dermal fibroblast therapy can be safely used to treat patellar tendinopathy, showing a faster short-term treatment response compared to plasma injection alone, with significant improvements in pain and function. A total of 48 patients were enrolled in the study, divided into two groups: one received injections of autologous dermal fibroblast plasma, and the other received injections of plasma alone. No serious adverse reactions or significant medical events were observed in either group after 24 weeks of treatment. VAS scores showed a downward trend in both groups, with the improvement being more pronounced in the cell therapy group. It preliminarily demonstrates the therapeutic value of dermal fibroblasts in the treatment of patellar tendinopathy (Clarke et al., 2011). This study suggests that the mechanism by which dermal fibroblast therapy acts on patellar tendinopathy mainly involves the differentiation of dermal fibroblasts into tenocytes and the production of collagen. Do fibroblasts possess the capacity to transdifferentiate into tenocytes *in vivo*? Using a zebrafish tendon injury model, Rajan et al. provided direct evidence to address this key question. The zebrafish model confirmed that fibroblasts can transdifferentiate into functional tenocytes following tendon injury, and this process is dependent on the Hedgehog signaling pathway (Rajan et al., 2023). Studies using zebrafish models have revealed *in vivo* evidence that fibroblasts can transdifferentiate into tenocytes after tendon injury, providing an important developmental biological basis for their cell plasticity. However, whether a similar transdifferentiation mechanism exists in mammalian systems remains to be further investigated.

### 3.2 Diseases of the skeletal system

#### 3.2.1 Fracture healing and bone defects

The treatment of bone defects, especially large bone defects, has been a major challenge due to the complexity of local injuries and the lack of ideal repair materials. The goal for everyone is to find an ideal bone substitute material that can achieve or approach the effects of autologous bone transplantation as closely as possible. Considering patient acceptance, operability, and simplicity, researchers have begun to focus on inducible osteogenic precursor cells (IOPC). Among them, dermal fibroblasts, which are widely distributed in the body, abundant in number, superficial in location, easy to obtain, easy to culture and pass on, and rapidly proliferate and divide, have become the focus of research.

The simplest and most common form of bone trauma is a fracture, and its healing process proceeds through four stages: inflammatory response, clearance, fibrous callus, and bony callus. Fibroblasts are abundant in the skeleton, and many studies have shown that fibroblasts affect normal fracture healing (Mascharak et al., 2021). Electron microscopy observations of the local bone formation area of fractures show that, in addition to osteoblasts playing an osteogenic role here, fibroblasts also exhibit similar osteogenic manifestations (Chai and Tang, 1979). For example, fibroblasts produce matrix vesicles, as do osteoblasts, and deposit calcium salts within the vesicles. Calcified matrix vesicles form tufted ball-like calcium nodules, which subsequently merge and fuse into bone tissue. This indicates that fibroblasts, like osteoblasts, possess the necessary conditions for providing calcium salt deposition and bone formation. Regarding the osteogenic effect of fibroblasts, some scholars believe that it results from the inherent characteristics of fibroblasts expressed under the specific conditions of fracture. That bone morphogenetic protein (BMP) in the bone matrix has a certain inductive effect on fibroblasts (Ta et al., 1981; Onishi et al., 1998).

The process of fracture healing involves various growth factors, mainly including BMPs, transforming growth factor-β (TGF-β), fibroblast growth factors (FGFs), and insulin-like growth factors (IGFs). They play roles in bone regeneration and repair, as well as in cell proliferation and differentiation (Li et al., 2016). Fibroblasts exhibit chemotaxis and are attracted to the fracture site by trauma stress and chemotactic factors (such as lymphokines), playing an important regulatory role in fracture healing (Li et al., 2024). Aloise et al. found that adding TGF-β1 to the osteogenic medium can increase the ALP activity and osteocalcin content in the supernatant of human dermal fibroblasts (Aloise et al., 2014). Dumic-Cule et al. found that under the action of BMP-2 or a combination of BMP-2 and TGF-β, fibroblasts can be converted into osteoblasts (Dumic-Cule et al., 2018).

As fibroblasts directly participate in the formation of fibrous callus during fracture healing and have the ability to be induced into bone, compared to other cells with osteogenic effects (such as periosteal osteoblasts and bone marrow stromal cells), dermal fibroblasts have advantages such as convenient sampling, minimal damage to the body, easy survival *in vitro* culture, and rapid proliferation and reproduction. They are expected to replace autologous or allogeneic bone grafts for the treatment of large bone defects that are difficult for the body to repair, opening a new and promising path for their repair and treatment.

Notably, although the plasticity of fibroblasts contributes to tissue repair, it can lead to pathological bone formation, namely, heterotopic ossification (HO), under specific conditions. Following trauma, burns or surgery, fibroblasts may abnormally differentiate into osteoblasts, resulting in the formation of ectopic bone in non-skeletal tissues (Li et al., 2025). This dual-edged sword effect alerts us to the potential risk of fibroblast therapy, namely, excessive osteogenesis, and indicates that targeted fibroblast treatment requires precise regulation to avoid aberrant osteogenic responses.

#### 3.2.2 Lumbar disc degeneration

Lumbar disc degeneration refers to the degeneration of lumbar discs, accompanied by common spinal diseases such as low back pain and limited lumbar function. Lumbar disc degeneration is considered a major cause of low back pain (Cheung et al., 2009). Both in China and globally, low back pain is the leading cause of loss of individual labor capacity (Global Burden of Disease Study 2017 Collaborators, 2018; Global Burden of Disease Study 2013 Collaborators, 2015).

Currently, treatments for degenerative lumbar disc disease include conservative treatment and surgical treatment. Conservative treatment methods include medication (mainly analgesics), bed rest, and physical therapy (such as massage, acupuncture, and moxibustion). If conservative treatment fails or neurological symptoms (decreased muscle strength, perineal numbness, urinary and fecal incontinence) occur, surgical treatment is necessary. Surgical treatment mainly includes percutaneous endoscopic lumbar discectomy (PELD) and interbody fusion surgery. The risk of recurrence after nucleus pulposus removal is high, and it may also lead to long-term loss of intervertebral height and segmental instability. Interbody fusion surgery, on the other hand, sacrifices lumbar spine mobility, altering biomechanical properties and increasing stress on adjacent intervertebral discs, accelerating degeneration (Harrop et al., 2008; Rihn et al., 2009).

The current treatment strategies aim to manage symptoms, primarily to alleviate pain, and conservative treatment cannot delay the progression of degeneration, with unclear long-term benefits for patients and poor quality of life. In response to this unmet clinical need, innovative cell therapies aimed at “regeneration” have shown some potential applications. The intervertebral disc is composed of the nucleus pulposus, the annulus fibrosus, and the cartilage end-plate. The degeneration process of the intervertebral disc begins with the degeneration of the nucleus pulposus tissue. Due to the lack of blood supply to the intervertebral disc, the microenvironmental oxygen content is low, nutrient supply is scarce, cell number is low, and metabolic rate is low. Therefore, activating regeneration and reversing intervertebral disc degeneration through cell therapy is a great challenge (Kandel et al., 2008). Dermal fibroblasts have low oxygen and nutrient demand and possess tissue repair capabilities, which have attracted widespread attention for the treatment of intervertebral disc degeneration.

Chen et al. isolated and cultured dermal fibroblasts from the dorsal skin of SD rats and the postauricular skin tissue of cynomolgus monkeys, respectively. He induced degeneration of the tail intervertebral disc of rats and the lumbar intervertebral disc of cynomolgus monkeys using acupuncture. He injected autologous fibroblasts into the degenerated intervertebral disc of the model animals via local injection. Imaging results showed that the height of the intervertebral disc was maintained after treatment with dermal fibroblasts, the intervertebral disc water content improved, overall spine stability was enhanced, osteophyte formation was reduced, and biomechanical strength increased. Histological results showed that a large amount of regular fibrous tissue was visible in the intervertebral disc treated with dermal fibroblasts, and the structure of the nucleus pulposus tissue was relatively intact. They believe that the mechanism by which fibroblasts treat intervertebral disc degeneration mainly involves inhibiting aseptic inflammation, as well as repairing degenerative intervertebral discs through fibrosis mediated by the secretion of extracellular matrix such as collagen and TGF-β, thereby providing structural support (Chen et al., 2020).

Peng I et al. used a rabbit intervertebral disc degeneration model to compare the effects of neonatal human dermal fibroblasts (nHDFs) and rabbit dermal fibroblasts (RDFs) transplantation on host immune response, disc height, and disc composition. The study showed that both nHDFs and RDFs can increase markers of disc regeneration (disc height, type I and type II collagen, proteoglycans) and inhibit disc inflammation. Furthermore, the human cells transplanted into the rabbit disc did not induce a higher immune response than the rabbit cells. These results support the idea that the intervertebral disc has immune privilege and can tolerate allogeneic or xenogeneic grafts (Shi et al., 2019).

## 4 Application challenges and prospects

### 4.1 Current challenge

Over the past few decades, significant progress has been made in the study of fibroblast biology and applications. These advancements have revealed similarities and unique features of fibroblasts across organs such as skin, lungs, heart, and skeletal muscle, which are currently being used to treat human diseases (Plikus et al., 2021). Dermal fibroblasts have become a focal point in disease treatment research due to their widespread distribution, ease of collection, minimal harm to the body, high survival rate *in vitro* culture, and rapid proliferation. Numerous studies have confirmed the therapeutic potential of dermal fibroblasts in the treatment of musculoskeletal diseases, but their clinical translation remains challenging.

Dermal fibroblasts do not constitute a homogeneous cell population. Their biological functions exhibit donor heterogeneity depending on the donor’s age, gender and health status. Meanwhile, dermal fibroblasts derived from different anatomical sites also display regional heterogeneity. For instance, retroauricular skin fibroblasts originate from ectodermal neural crest cells, while foreskin fibroblasts are derived from mesodermal mesenchymal cells. Fibroblasts with distinct embryonic origins and anatomical locations differ in both identification biomarkers and biological functions (Torregrossa et al., 2025). Accordingly, the absence of standardized cell manufacturing protocols and quality control systems may result in inconsistent quality and therapeutic efficacy of cell products across different batches. It is necessary to establish a standardized donor harvesting protocol and clarify screening criteria regarding donors’ age, gender, disease status, and other indicators; formulate standardized manufacturing procedures and quality testing specifications; define the functional cell subpopulations; narrow the detection scope of biological activity; and strengthen the overall quality control of cells.

Unlike the targeted nature of gene therapy, most cell therapy products still fail to identify the key functional factors and clarify their therapeutic targets to date, resulting in inconsistent and variable treatment efficacy. Although dermal fibroblasts are known to have paracrine and immunomodulatory effects, but the key factors that underlie their function remain unclear. There is a lack of reliable biomarkers for real-time monitoring of cell activity and efficacy *in vivo*, making it difficult to determine the optimal treatment window and cell injection dose.

Fibroblasts serve as the main force of tissue repair and also act as the primary driver of fibrotic scar formation. In musculoskeletal diseases, excessive or persistent activation of fibroblasts leads to the deposition of functionally inferior fibrotic tissue, rather than the regeneration of tissue with normal mechanical properties (Kili and Kocaefe, 2026). Most current studies remain at the animal experiment and early clinical stages, with a severe shortage of high-quality randomized controlled clinical trials. The long-term safety of fibroblasts has not been fully evaluated, including their tumorigenic potential, abnormal differentiation (such as heterotopic ossification caused by osteogenic differentiation of fibroblasts), immunogenicity, and the risk of long-term fibrosis. Although dermal fibroblasts lowly express MHC class II antigens and therefore carry a low risk of immunotoxicity, fibroblasts possess strong proliferative capacity *in vitro*. Thus, close attention should still be paid to whether abnormal proliferation occurs after *in vivo* cell transplantation. Dermal fibroblasts have been widely used in the treatment of musculoskeletal diseases, and their administration is mostly via local injections, such as tendon injections. However, there may be problems, such as difficulty with cell adhesion and low retention, which require the use of biological materials (such as hydrogels) to improve cell survival and colonization efficiency. Furthermore, fibroblasts act as sensitive receptors of mechanical signals. Tenocytes in tendons and ligaments are extremely responsive to mechanical loading: moderate stretching promotes ECM synthesis, whereas excessive or insufficient loading induces catabolism. However, the mechanical environment at injury sites is often imbalanced. There remains a lack of in-depth understanding of how transplanted cells adapt to and integrate into this dynamically changing microenvironment.

### 4.2 Future direction

Precisely regulating the functional state of fibroblasts will be the core direction to break through the bottleneck. Gene editing of dermal fibroblasts to enhance their specific functions and develop “enhanced” cell therapy drugs. Such as overexpression of osteogenic and tendonogenic factors (BMP, TGF-β), improving the directed secretion ability of fibroblasts, or introducing the IL-10 gene to enhance anti-inflammatory ability, and introducing the VEGF gene to enhance angiogenic ability. Kim et al. used two transcription factors, Oct4 and Cbfβ, to directly convert human dermal fibroblasts into functional osteoblasts (piOBs). Transplantation of piOBs into a mouse calvarial defect model induced extensive new bone formation, demonstrating the *in vivo* therapeutic efficacy (Kim et al., 2026).

Exosomes are nanoscale extracellular vesicles that play a crucial role in intercellular communication due to their content of bioactive substances such as RNA and proteins. Dermal fibroblasts have abundant paracrine functions and can secrete hundreds of cytokines and growth factors. In recent years, exosomes have emerged as a promising tool in tissue regeneration, potentially marking a new trend in cell-free therapy.

Dermal fibroblasts are widely used in the treatment of musculoskeletal diseases, and cell survival and retention time are crucial to their efficacy. Developing novel biomaterials as “carriers” for fibroblasts can provide attachment points for the cells, thereby extending their survival and retention. For example, dermal fibroblasts can be encapsulated in temperature-sensitive, photocrosslinked hydrogels and injected into the lesion site to form a three-dimensional scaffold; scaffolds with biomimetic structures can be constructed using 3D printing technology and then loaded with cells for transplantation to the affected area.

With advances in science, more and more technologies are being applied in biomedicine. By utilizing technologies such as single-cell sequencing, proteomics, and secretomics, we can deeply analyze the characteristics and functions of different subpopulations of dermal fibroblasts and conduct in-depth mechanistic studies to identify biomarkers that exert therapeutic effects. Based on the disease type, severity, and individual patient characteristics, select the most suitable dermal fibroblast subpopulation for disease treatment. Strengthen the collection of real-world study (RWS) data to verify its safety and effectiveness in human musculoskeletal diseases, laying the foundation for large-scale application.

## 5 Summary

Musculoskeletal diseases are a major medical burden affecting the health of hundreds of millions of people worldwide, encompassing a variety of pathological conditions such as tendon injury, osteoarthritis, non-union fracture, intervertebral disc degeneration, and ligament tear. A common feature of these diseases is the limited self-repair capacity of affected tissues, and conventional therapeutic approaches are difficult to achieve genuine structural regeneration. In recent years, the rapid advancement of cell therapy strategies has opened up a new avenue for the treatment of musculoskeletal disorders. Dermal fibroblasts have shown great potential in the biological treatment of musculoskeletal diseases due to their convenient source, strong proliferative capacity, and rich paracrine function. Whether promoting tendon regeneration, cartilage repair, accelerating fracture healing, or restoring the biomechanical function of intervertebral discs, they all play a role through various mechanisms, such as regulating the microenvironment, promoting tissue regeneration, and inhibiting inflammation. Despite the current challenges of cellular heterogeneity, unclear mechanisms of action, and safety concerns, the development of cell engineering technology and regenerative medicine is expected to make dermal fibroblasts a new and highly effective biotherapeutic, opening new avenues for the treatment of musculoskeletal diseases.
